# Radiomics analysis using magnetic resonance imaging of bone marrow edema for diagnosing knee osteoarthritis

**DOI:** 10.3389/fbioe.2024.1368188

**Published:** 2024-06-12

**Authors:** Xuefei Li, Wenhua Chen, Dan Liu, Pinghua Chen, Pan Li, Fangfang Li, Weina Yuan, Shiyun Wang, Chen Chen, Qian Chen, Fangyu Li, Suxia Guo, Zhijun Hu

**Affiliations:** ^1^ Longhua Hospital Affiliated to Shanghai University of Traditional Chinese Medicine, Shanghai, China; ^2^ Institute of Chinese Materia Medica, Shanghai University of Traditional Chinese Medicine, Shanghai, China

**Keywords:** radiomics, bone marrow edema, knee osteoarthritis, nomogram, magnetic resonance imaging

## Abstract

This study aimed to develop and validate a bone marrow edema model using a magnetic resonance imaging-based radiomics nomogram for the diagnosis of osteoarthritis. Clinical and magnetic resonance imaging (MRI) data of 302 patients with and without osteoarthritis were retrospectively collected from April 2022 to October 2023 at Longhua Hospital affiliated with the Shanghai University of Traditional Chinese Medicine. The participants were randomly divided into two groups (a training group, n = 211 and a testing group, n = 91). We used logistic regression to analyze clinical characteristics and established a clinical model. Radiomics signatures were developed by extracting radiomic features from the bone marrow edema area using MRI. A nomogram was developed based on the rad-score and clinical characteristics. The diagnostic performance of the three models was compared using the receiver operating characteristic curve and Delong’s test. The accuracy and clinical application value of the nomogram were evaluated using calibration curve and decision curve analysis. Clinical characteristics such as age, radiographic grading, Western Ontario and McMaster Universities Arthritis Index score, and radiological features were significantly correlated with the diagnosis of osteoarthritis. The Rad score was constructed from 11 radiological features. A clinical model was developed to diagnose osteoarthritis (training group: area under the curve [AUC], 0.819; testing group: AUC, 0.815). Radiomics models were used to effectively diagnose osteoarthritis (training group,: AUC, 0.901; testing group: AUC, 0.841). The nomogram model composed of Rad score and clinical characteristics had better diagnostic performance than a simple clinical model (training group: AUC, 0.906; testing group: AUC, 0.845; *p* < 0.01). Based on DCA, the nomogram model can provide better diagnostic performance in most cases. In conclusion, the MRI-bone marrow edema-based radiomics-clinical nomogram model showed good performance in diagnosing early osteoarthritis.

## Introduction

Osteoarthritis (OA) is a degenerative disease characterized by persistent pain and joint dysfunction (Hawker, 2019). According to statistics, >500 million people suffer from OA worldwide (Quicke et al., 2022). The pathological changes associated with OA are complex, and cartilage loss has traditionally been considered a key feature in OA (Yunus et al., 2020; Wang et al., 2022). However, whether the initial pathological changes in OA originate from subchondral bone, calcified cartilage, or cartilage remains controversial. Under physiological conditions, osteochondral units comprising noncalcified cartilage, calcified cartilage, subchondral cortical bone, and subchondral trabecular bone adeptly transfer loads and provide structural support. Pathological changes in any tissue structure in the functional unit destroy the integrity of the joint mechanism and result in the loss of its physiological function. However, cartilage and subchondral bone exhibit different mechanical adaptabilities. Stress distribution in the cartilage changes with the expansion of subchondral bone (Li et al., 2024). Even a slight 1%–2% increase in subchondral-bone size substantially amplifies stress on the cartilage (Burr and Gallant, 2012). Under normal physiological conditions, subchondral bone effectively absorbs mechanical loads, maintaining joint function and overlying cartilage stability. The contribution of pathological changes in the subchondral bone to OA progression has attracted interest (Zhang H. et al., 2023). Pathological changes in the subchondral bone include bone marrow edema-like lesions and bone cysts (Hu et al., 2021). Bone marrow edema-like lesions fundamentally participate in the progression of OA, considered a basic risk factor for pathological structural changes and the most common imaging manifestations (Driban et al., 2022).

In preclinical experimental studies, subchondral bone marrow edema occurred during or before cartilage loss (Zhang et al., 2018). Clinical studies have found a strong correlation between bone microstructural changes in bone marrow edema and the pathological characteristics of cartilage structure and volume loss in the human tibial plateau (Kon et al., 2016; Zhang S. et al., 2023). In addition, OA-related pain is closely associated with bone marrow edema (Perry et al., 2019; Koushesh et al., 2022). A better understanding of the relationship between bone marrow edema and OA can provide more information for the diagnosis, progression, and clinical management of diseases.

The most sensitive imaging method for evaluating OA is magnetic resonance imaging (MRI) (Demehri et al., 2023). Wilson et al. (1988) first localized and detected areas with increased signal strength in the tibia and femur of patients with OA by using an enhanced magnetic-resonance sequence (Wilson et al., 1988). Nevertheless, histological analysis, until 2010, disclosed that bone-marrow edema encompasses marrow fibrosis, vascular shifts, and local fat necrosis caused by trabecular microfractures (Leydet-Quilici et al., 2010a). Therefore, these pathological changes are referred to as subchondral bone marrow lesions (SBMLs). On MRI scans, bone marrow edema is identified as a high-signal area on T2-weighted fat saturation images (Kostopoulos et al., 2023). MRI signal intensity, volume, and shape parameters of bone marrow edema are considered biomarkers of joint pain, dysfunction, and the severity of cartilage damage (Gong et al., 2016; Dong et al., 2017; Deng et al., 2021). However, these assessment methods are time-consuming and subjective, with poor intra-observer and inter-observer variability. In contrast, radiomics extracts a large number of quantitative image features, such as texture, intensity, and geometric shape from conventional images, noninvasively captures subtle lesions, and provides the possibility for developing new image-based diagnostic methods (Kumar et al., 2012). Recently, researchers have evaluated radiomics features using MRI to evaluate knee OA. Hirvasniemi et al and Xue et al used MRI-based radiomics features from the subchondral bone to identify knee OA. However, the extraction site of the radiomics features is not detailed in the area of bone marrow edema (Hirvasniemi et al., 2021; Xue et al., 2022). Since bone marrow edema may be the first pathological change in OA and participate in its pathological progression, we speculated that a predictive model constructed from radiomics information extracted from the bone marrow edema region may improve diagnostic sensitivity. Therefore, this study aimed to create a diagnostic model for knee OA based on radiomics of bone marrow edema using MRI.

## Materials and methods

### Patients

**FIGURE 1:**
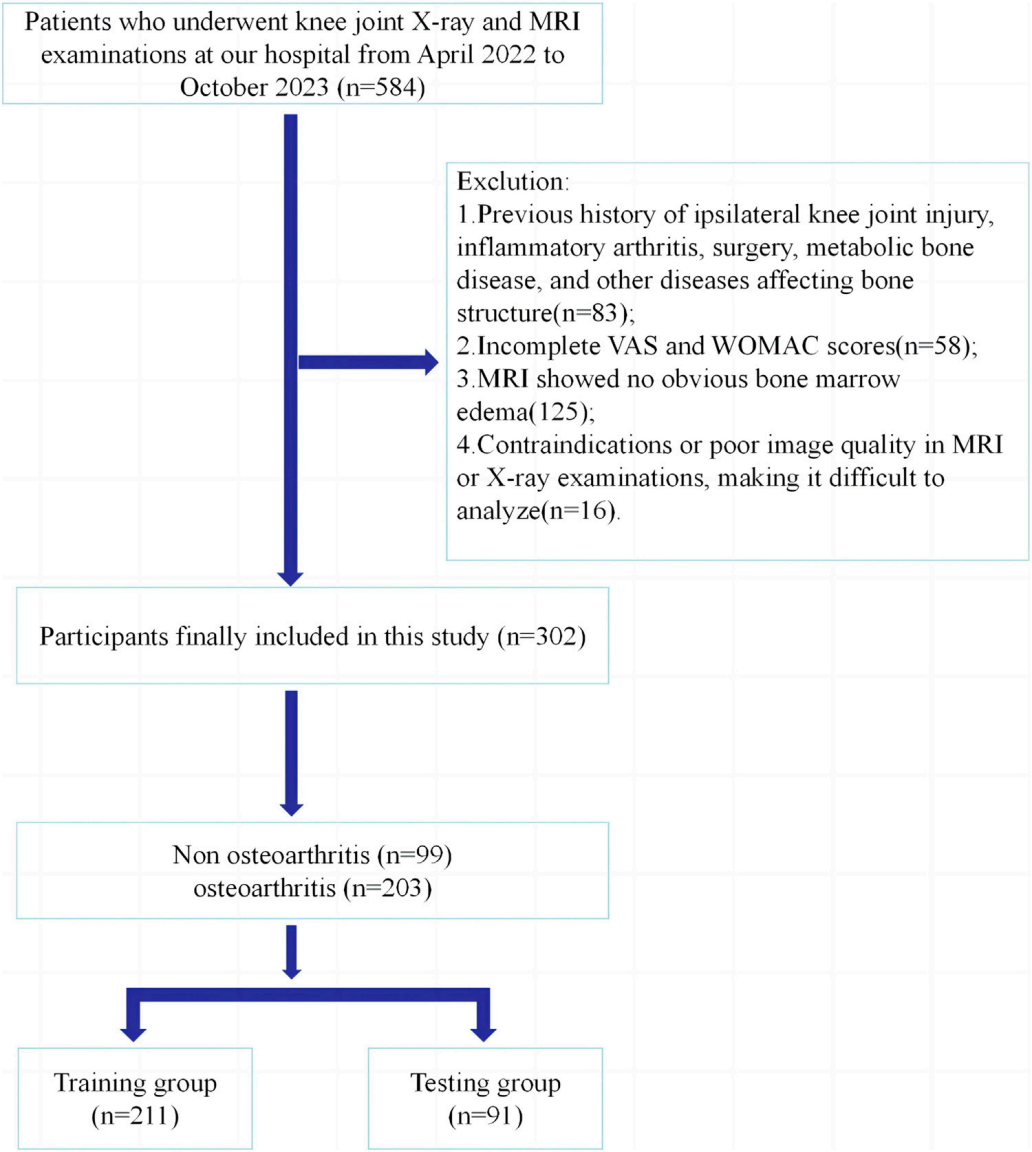
The process of participant registration.

We reviewed the radiology databases of Longhua Hospital (affiliated with the Shanghai University of Traditional Chinese Medicine). Participants underwent knee joint radiography and MRI examinations at our hospital between April 2022 and October 2023. The inclusion criteria were: patients who underwent knee joint radiography and MRI examination in our hospital, with the latter revealing bone marrow edema; and those who completed the standard visual analog scale (VAS) and Western Ontario and McMaster Universities Arthritis Index (WOMAC). The exclusion criteria were: a history of knee degenerative OA, inflammatory arthritis, osteoporosis, and other diseases that affect bone structure; And the contraindications or poor image quality of MRI or radiographic examination make it difficult to analyze. This was a retrospective study, and the requirement for informed consent was waived. The study protocol was approved by the hospital’s Ethics Committee. Figure 1 shows the process of participant registration.

### Evaluation of knee OA

Two senior orthopedic physicians at our hospital diagnosed and evaluated knee OA based on clinical symptoms, physical examination, and imaging manifestations of the patients. When there was a dispute over the results, a third senior orthopedic physician arbitrated.

The diagnostic criteria for knee OA were as follows (Zhang et al., 2010; Joint Surgery Branch of the, 2021): 1) Recurrent knee pain within 1 month; 2) Knee joint dysfunction with occasional bone fricatives during movement; 3) Kellgren–Lawrence (K–L) grade ≥2 in knee joint radiography.

### MRI scanning technology

All participants underwent an MRI examination. MRI scans were performed using a 3T MRI unit (Verio; Siemens Healthineers, Erlangen, Germany) with an 8-channel phased array knee coil. Sagittal 2D fast spin-echo proton density-weighted sequences with fat suppression were used to evaluate bone marrow edema and cartilage injury (repetition time/echo time, 2400/43; field of view, 100 mm; matrix, 320 × 320; flip angle, 150°; and section thickness, 3.5 mm).

### Image segmentation

The area of subchondral bone marrow edema was the target of image segmentation. In sagittal 2D fast spin-echo proton density-weighted sequences with fat suppression, areas of bone marrow edema were delineated as regions of interest (ROI) in each layer. Image segmentation was independently performed by two radiologists (A and B). Participants were unaware of whether they had been diagnosed with knee OA. The open-source software 3D Slicer 4.11.0 (https://www.slicer.org/) was used for ROI segmentation, which was completed by radiologist A. Radiologist B reviewed all manually segmented ROIs by Radiologist A. If there is a dispute between radiologist A and radiologist B regarding the delineation range of bone marrow edema, radiologist C shall arbitrate. Supplementary Figure S1 shows the workflow of radiomics analysis in this study and presents a schematic diagram of ROI segmentation.

### Radiomics feature extraction and selection

All radiomics features were extracted from each ROI of bone marrow edema using Pyradiomics (https://pyradiomics.readthedocs.io/en/latest/). Typically, radiomic features include three categories: intensity, texture, and geometry. We used different methods such as the gray-level size zone matrix (GLSZM), gray-level co-occurrence matrix (GLCM), gray-level run length matrix (GLRLM), and neighborhood gray-tone difference matrix (NGTDM) to extract texture features.

We selected features in three steps. First, we screened features using the T-test or Mann–Whitney U test. Only radiological features with *p* < 0.05 were retained. Second, we used Spearman’s rank correlation coefficient to calculate the correlation between highly repetitive features, while retaining features with correlation coefficients>0.9. Finally, the least absolute shrinkage and selection operator (LASSO) regression model was used to construct the signature of the dataset. A 10-fold cross-validation with minimum criteria was employed, where the final value of λ yielded the minimum cross-validation error. The retained features with nonzero coefficients were used for regression model fitting and were combined into a radiomics signature to obtain the radiomics score.

### Radiomics model construction

We input the final features (after LASSO feature selection) into the machine learning model, including a support vector machine (SVM) and logistic regression (LR) (seven types) for model construction. To evaluate the diagnostic performance of the predictive model, we plotted a receiver operating characteristic (ROC) curve and analyzed the area under the curve (AUC), diagnostic specificity, sensitivity, negative predictive value (NPV), positive predictive value (PPV), precision, and F1.

### Clinical characteristics model construction

Age, X-ray K–L grading, and WOMAC were selected as the clinical characteristics for the diagnosis of knee OA. The selected clinical characteristics were used to construct a clinical characteristics model. The construction process of the clinical characteristics model was almost identical to that of the radiomic signatures.

### Radiomic nomogram construction

A radiology nomogram was established by combining clinical characteristics and radiomics signatures. We calculated a calibration curve to compare the consistency between the predicted and actual observed values. We quantified the distinguishability of the nomogram by calculating the AUC of two groups, and evaluated the clinical utility of the nomogram using Mapping Decision Curve Analysis (DCA).

### Statistical analysis

We used Fisher’s exact test or the Chi-squared test to analyze categorical variables, and the T-test or Mann–Whitney U test was applied for continuous variables. All statistical analyses were conducted using the Statsmodes package for Python (version 0.13.2; Python Software Foundation, Wilmington, DE, USA). Statistical significance was set at *p* < 0.05.

## Results

### Comparison of clinical characteristics

The clinical features of patients with OA and non-OA in the training and independent testing groups are presented in Tables 1, 2. Patients of 65.89% (199/302) were women, and the average age of all patients were 63.34 ± 9.51 years. According to clinical diagnosis, there were 203 OA patients and 99 non-OA patients. The OA patients constituted 67.77% and 65.93% in the training (N = 211) and testing (N = 91) groups, respectively.

**TABLE 1:** Clinical characteristics of participants in our cohort.

Characteristic	Total(n = 302)	Non-OA(n = 99)	OA(n = 203)	p value
age	63.34±9.51	61.00±10.38	64.49±8.86	0.004
WOMAC	109.68±19.51	100.03±20.75	114.39±17.04	<0.001
gender				0.012
Female	199(65.89)	55(55.56)	144(70.94)	
male	103(34.11)	44(44.44)	59(29.06)	
X-ray K-L grading				<0.001
0	64(21.19)	58(58.59)	6(2.96)	
1	27(8.94)	6(6.06)	21(10.34)	
2	60(19.87)	10(10.10)	50(24.63)	
3	93(30.79)	15(15.15)	78(38.42)	
4	58(19.21)	10(10.10)	48(23.65)	

OA: osteoarthritis; WOMAC: Western Ontario and McMaster Universities Arthritis Index(0-10 points per piece); K-L: Kellgren-Lawrence

**TABLE 2:** Clinical characteristics of participants in the training and testing groups.

Comparative analysis of different radiomics models												
model_name	Accuracy	AUC	95% CI	Sensitivity	Specificity	PPV	NPV	Precision	Recall	F1	Threshold	Task
LR	0.725	0.696	0.6158–0.7769	0.937	0.279	0.732	0.679	0.732	0.937	0.822	0.639	label-train
LR	0.747	0.822	0.7353–0.9088	0.900	0.452	0.761	0.700	0.761	0.900	0.824	0.621	label-test
SVM	0.768	0.901	0.8512–0.9518	0.993	0.894	0.747	0.952	0.747	0.993	0.853	0.708	label-train
SVM	0.681	0.841	0.7589–0.9239	0.950	0.861	0.687	0.625	0.687	0.950	0.797	0.665	label-test
KNN	0.758	0.802	0.7455–0.8594	0.937	0.382	0.761	0.743	0.761	0.937	0.840	0.800	label-train
KNN	0.736	0.745	0.6444–0.8454	0.917	0.387	0.743	0.706	0.743	0.917	0.821	0.800	label-test
RandomForest	0.995	1.000	0.9995–1.0000	1.000	0.985	0.993	1.000	0.993	1.000	0.997	0.600	label-train
RandomForest	0.725	0.748	0.6390–0.8578	0.783	0.613	0.797	0.594	0.797	0.783	0.790	0.600	label-test
ExtraTrees	1.000	1.000	1.0000–1.0000	1.000	1.000	1.000	1.000	1.000	1.000	1.000	1.000	label-train
ExtraTrees	0.780	0.802	0.7067–0.8965	0.867	0.613	0.812	0.704	0.812	0.867	0.839	0.600	label-test
XGBoost	0.991	1.000	1.0000–1.0000	1.000	0.971	0.986	1.000	0.986	1.000	0.993	0.680	label-train
XGBoost	0.747	0.796	0.7050–0.8874	0.817	0.613	0.803	0.633	0.803	0.817	0.810	0.648	label-test
MLP	0.716	0.763	0.6922–0.8342	0.951	0.221	0.720	0.682	0.720	0.951	0.819	0.672	label-train
MLP	0.703	0.796	0.7033–0.8891	0.917	0.290	0.714	0.643	0.714	0.917	0.803	0.727	label-test

### Feature selection and rad-score establishment

**FIGURE 2:**
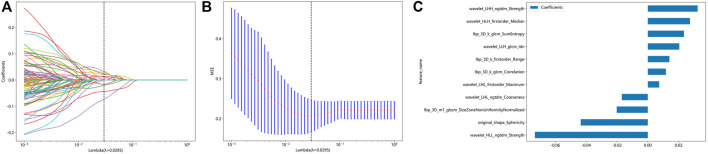
Radiomic feature selection based on LASSO algorithm and Rad score establishment. **(A** and **B)** Ten-fold cross-validated coefficients and 10-fold cross-validated MSE. **(C)** The histogram of the Rad score based on the selected features.

After extractiong, 1,384 radiomic features were obtained. Finally, 11 features with nonzero coefficients obtained after screening were established. Figures 2A,B show the coefficients and mean standard error (MSE) for the 10x validation, Figure 2C shows the coefficient values of the final selected nonzero features. The formula for calculating the rad score is shown in Supplementary Material S1.

**FIGURE 3:**
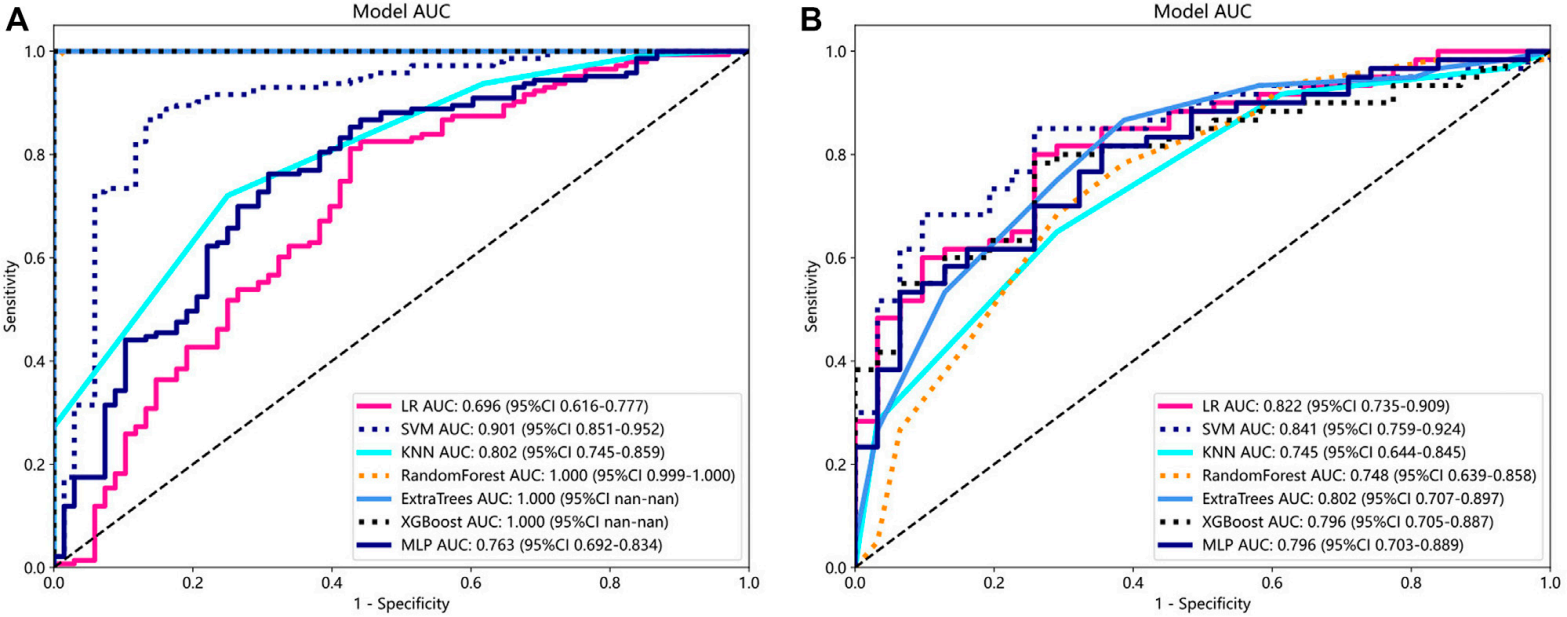
Comparison of radiometric feature model predictions for the training **(A)** and testing groups **(B)**. SVM achieved the best performance in both the training and testing groups.

To determine the best-performing model, we constructed seven models, including SVM, LR, and KNN. Compared to the other models, the SVM model exhibited the best performance. Supplementary Material S2 displays the information for all models. The SVM model achieved the best AUC for the training and test cohorts, reaching 0.901 and 0.841 for the diagnosis of knee OA, respectively. Therefore, SVM was used as the basic model for constructing clinical features. Figure 3 shows a comparison of the radiomics features between the different models in the training and testing groups.

### Comparison of clinical, radiomic, and nomogram models

**FIGURE 4:**
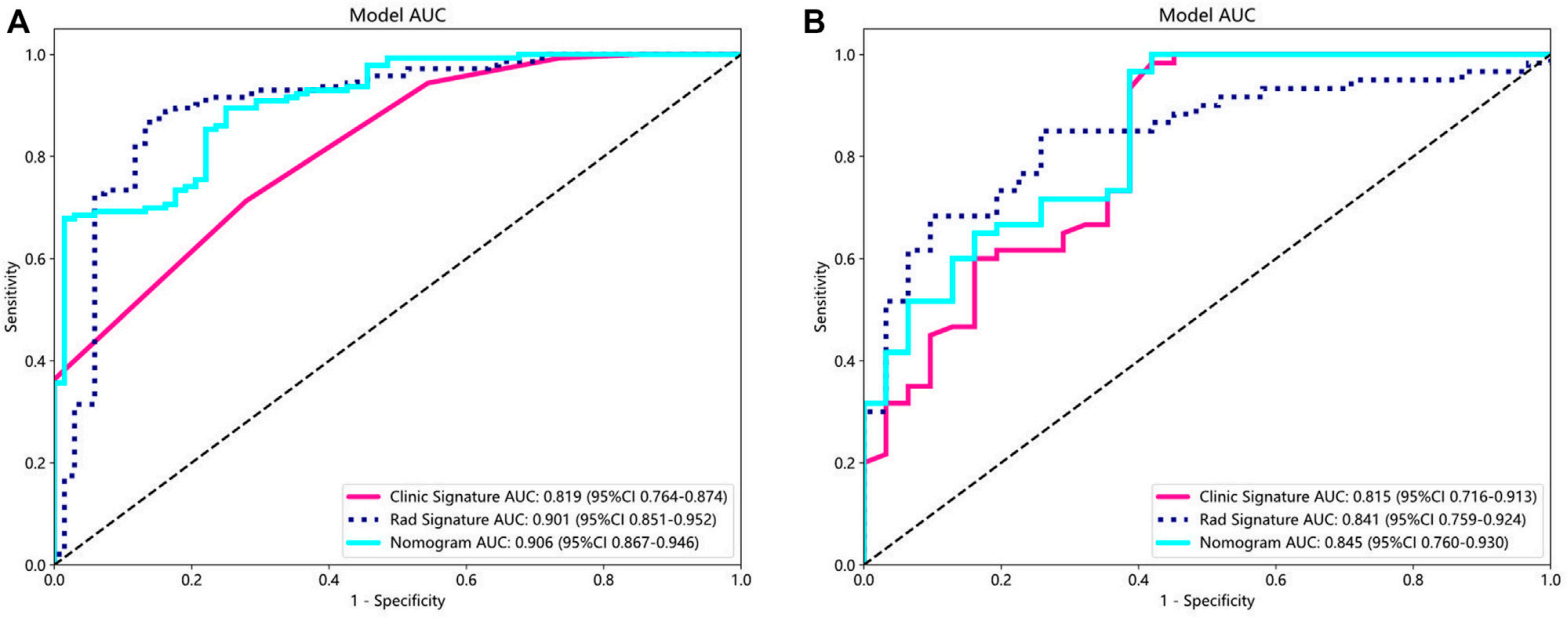
AUC comparison of clinical, radiological, and nomogram models in the training **(A)** and testing **(B)** groups. The combined nomogram performed optimally in both the training and testing cohorts.

For the clinical characteristic models, in the training group, the AUC value was 0.819 (95% confidence interval [CI], 0.764–0.874), and in the testing group, the AUC value was 0.815 (95% CI, 0.716–0.913). For the radiomics feature models, both the training group (AUC, 0.901; 95% CI, 0.851–0.952) and the testing group (AUC, 0.841; 95% CI, 0.759–0.924) had better AUC than the clinical model. The nomogram model showed good performance in both the training group (AUC, 0.906; 95% CI, 0.867–0.946) and the testing group (AUC, 0.845; 95% CI, 0.760–0.930) (Figure 4). In addition, we used the DeLong test (Supplementary Material S3) to compare radiomic signatures, clinical signatures, and nomograms. In the training and testing groups, the AUC of the nomogram model was significantly different from that of the clinical model (*p* < 0.01).

**FIGURE 5:**
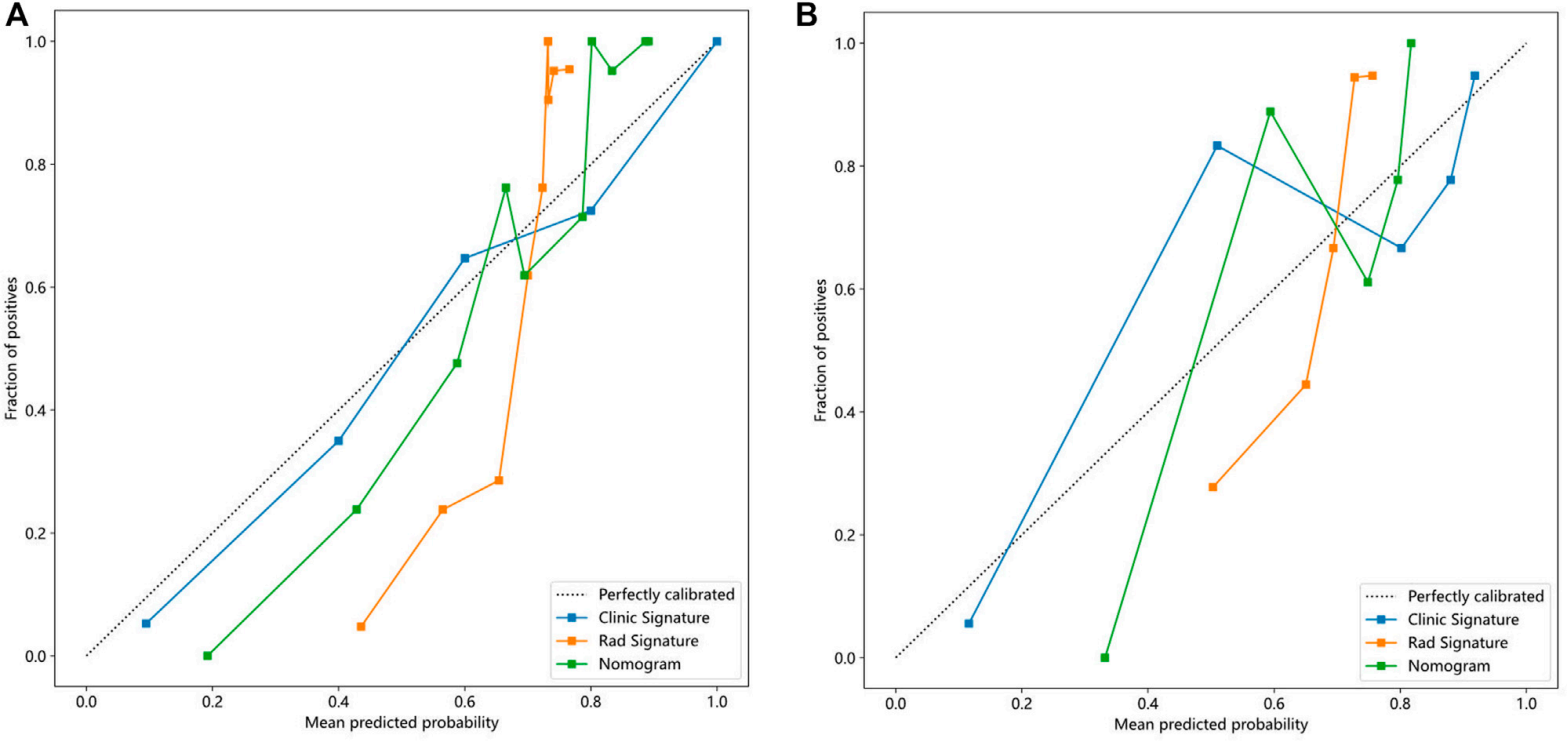
Calibration curves in the training and testing cohorts showing that the nomogram fits perfectly well in both the training **(A)** and testing groups **(B)**.

In addition, Figure 5 shows the nomogram calibration curve, Figure 5A, calibration curve of the radiomics nomogram in the training group. The Hosmer-Lemeshow test indicated that the difference was nonsignificant (*p* = 0.282). Figure 5B, calibration curve of the radiomics nomogram in the test group. The Hosmer-Lemeshow test also indicated that the results were nonsignificant (*p* = 0.267). The nomogram calibration curves are based on the agreement between the probability of the diagnosing knee osteoarthritis and the actual observation results. Supplementary Material S4 is the Hosmer Lemeshow H test.

**FIGURE 6:**
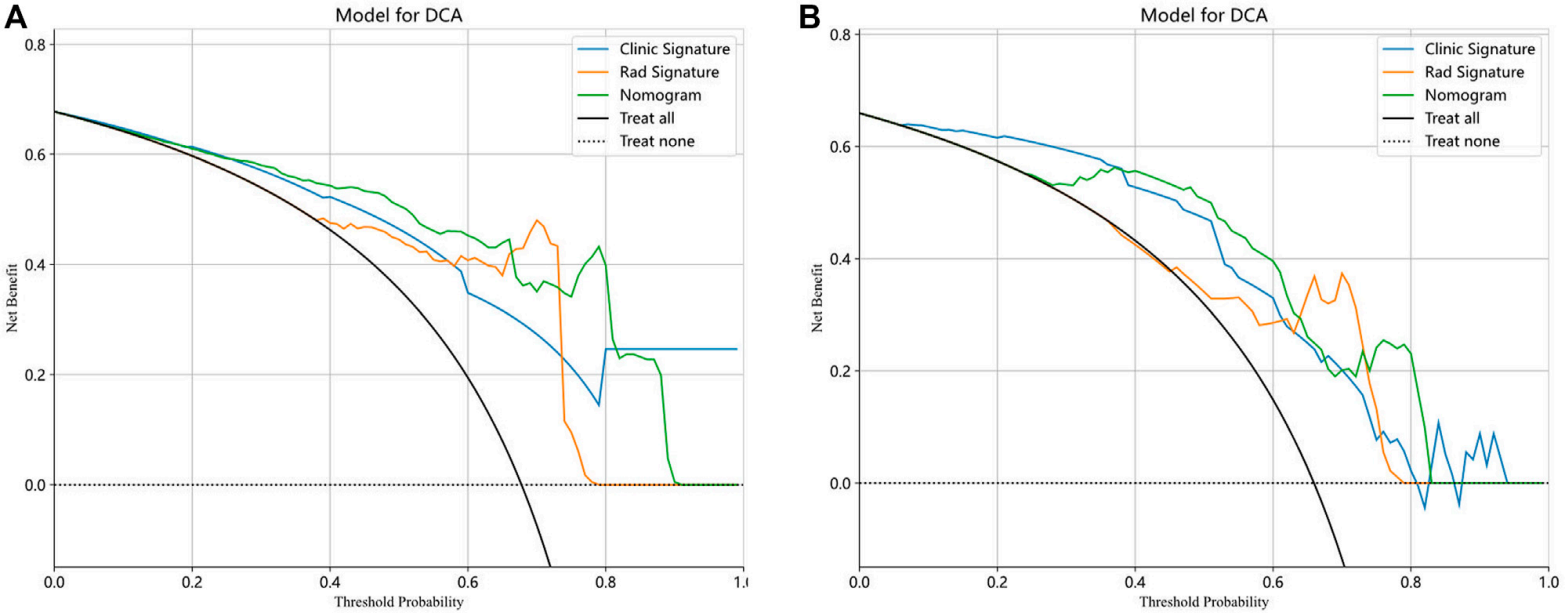
The decision curve analysis (DCA) of the three models of the training **(A)** and testing **(B)** groups.

**FIGURE 7:**
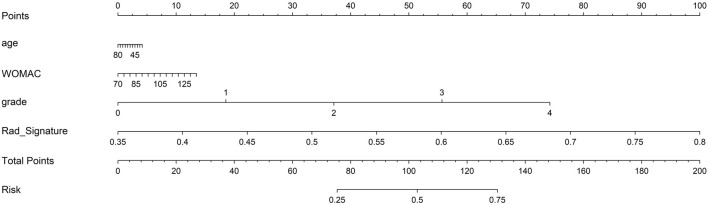
The clinical application of nomogram in the diagnosis of osteoarthritis.

Finally, each model was evaluated using DCA. Based on the DCA, among these three models, the nomogram model is higher than the other two within a large threshold range, indicating that the nomogram model has significant advantages (Figure 6). Figure 7 shows the nomogram developed to visualize the combined model and reflect the diagnosis of OA.

## Discussion

In this study, we developed a comprehensive model that included the rad-score, age, X-ray K–L grading, and WOMAC functional score, and established a diagnostic model for knee OA based on subchondral bone marrow edema. The nomogram model showed the best discriminative ability and fit, indicating a good predictive and diagnostic performance. The AUC values of the training and test groups were 0.986 and 0.845, respectively.

Bone marrow edema-like lesions fundamentally participate in the progression of OA and are considered basic risk factors for pathological structural changes and are the most common imaging manifestations (Driban et al., 2022). The main manifestation is low signal abnormality of the subchondral bone displayed on T1 weighted images and high signal abnormality of the subchondral bone displayed on T2 weighted images (Chimenti et al., 2020). Wilson et al. (1988) first localized and detected areas with increased signal strength in the tibia and femur of patients with OA using an enhanced magnetic resonance sequence (Wilson et al., 1988). However, the specific pathological changes associated with bone edema remain unclear. Until 2010, histological analysis revealed that bone marrow edema encompassed marrow fibrosis, vascular shifts, and local fat necrosis caused by trabecular microfractures (Leydet-Quilici et al., 2010b). Therefore, these pathological changes are referred to as subchondral bone marrow lesions (SBMLs). SBMLs are beneficial in the early screening and diagnosis of OA and is a determining factor for pain and the progression of OA. Joint cartilage injury is considered a typical pathological change in OA, and patients with bone marrow edema experience cartilage injury eight times more frequently than those without bone marrow edema (Horga et al., 2020; Peng et al., 2021). Several longitudinal studies have also found positive correlations between BML severity and cartilage defects, cartilage volume loss, joint space narrowing, and joint replacement (Fan et al., 2021; Li et al., 2022). Compared to the tibiofemoral joint, bone marrow edema and cartilage injury occur earlier and more frequently in the patellofemoral joint, and bone marrow edema is an indirect sign of cartilage injury (Dong et al., 2017), which is an important diagnostic value in predicting the occurrence and development of OA (Luo et al., 2023). Distinctive subchondral bone pathology marks the anteromedial OA-BML region, featuring subchondral bone plate thickening, heightened porosity, increased bone volume percentage, thicker trabeculae, reduced separation, focal sclerosis, fewer rod-shaped trabeculae and more plate-shaped trabeculae (Muratovic et al., 2019). OA-related pain is closely related to BML, and patients with knee OA pain are 2–5 times more likely to have BME than those without pain (Alliston et al., 2018). One study found a significant correlation between bone marrow edema and cold knee joint pain, and the degree of pain was positively correlated with the grading of bone marrow edema (Deng et al., 2021). In addition, some scholars have used the Boston Leeds Osteoarthritis Knee Score to score synovial, effusion, and bone marrow edema in patients with knee OA under weight-bearing conditions and found that BML and synovial effusion scores are highly correlated with weight-bearing knee joint pain (Lo et al., 2009; Perry et al., 2020). Koushesh et al. found that excessive blood vessels and innervation in BMLs contributed to our understanding of the relationship between BMLs and OA-related pain (Koushesh et al., 2022). Microarray analysis has demonstrated that the BML is a highly metabolically active region with increased cellular renewal, neuronal and bone remodeling, and inflammatory gene characteristics (Kuttapitiya et al., 2017).

The prediction and diagnosis of early OA have always been the focus of clinical orthopedic doctors. Patient symptoms, physical examination, and imaging are noninvasive methods for clinical OA diagnosis. However, ordinary radiographic recognition of changes in the bone structure and joint space indicates that obvious clinical symptoms have already appeared in OA (Amin et al., 2005). In contrast, magnetic resonance imaging can detect changes in bone structure and soft tissue around joints, particularly bone marrow edema, which can only be detected in magnetic resonance imaging (Guermazi et al., 2011). Predicting radiological narrowing and erosion of the joint spaces is of great significance (Haugen et al., 2016). However, there are currently no reports of MRI-based bone marrow edema as a predictor of OA. One study used radiomic features of the subchondral bone and trabecular structure parameters to construct a model for identifying radiological OA. The model constructed using radiomics features had a good recognition rate (AUC, 0.961) (Hirvasniemi et al., 2021). In another study, a combination model based on the MRI radiological features of the tibia and baseline features showed good radiological OA diagnostic performance (AUC, 0.80). However, as the most complex weight-bearing joint, pathological changes in the femur and tibia can lead to the occurrence of OA (Xue et al., 2022). In another study based on X-ray radiomics features and age-based diagnosis of knee OA, a nomogram model combining radiomics features and age showed good performance in accurately diagnosing OA (AUC, 0.849). However, this study focused on X-rays and could not predict early OA in the future (Li et al., 2023). Some studies have focused on radiomic analysis of joint-specific tissues to predict and diagnose OA. One study delineated the ROI of the cartilage to construct a model for diagnosing clinical OA. The radiomics feature model performed well in diagnosing clinical OA (AUC, 0.984) (Cui et al., 2023). In addition, a recent study suggested that the texture of the infrapatellar fat pad based on MRI is related to the future development of knee OA and can be used to predict the diagnosis of knee arthritis 1 year later (Ye et al., 2023). However, pathological changes in the subchondral bone are considered the first pathological changes in OA, and bone marrow edema on MRI is a typical imaging manifestation of pathological changes in the subchondral bone (Driban et al., 2022). Developing a predictive model for early OA that targets bone marrow edema would be beneficial for the early diagnosis of clinical OA. However, to the best of our knowledge, no relevant radiomics model is currently available.

In our study, a nomogram was constructed using Rad scores and clinical characteristics. The AUC of the radiomic features for diagnosing OA were 0.901 (training group) and 0.841 (testing group). The AUC for diagnosing the clinical characteristics of OA were 0.819 (training group) and 0.815 (testing groups), respectively. Nomograms constructed based on radiological and clinical characteristics showed good diagnostic performance for OA. The AUC values of two groups were 0.906 (training group: 95% CI, 0.867–0.946) and 0.845 (testing group: 95% CI, 0.760–0.930), respectively. The nomogram was effective in diagnosing OA in two groups, exceeding the diagnostic accuracy of single model. The decision curve indicates that if the threshold probabilities of patients are 0%–80% (training group) and 35%–83% (testing group), the radiological nomogram has better diagnostic value.

Based on our limited knowledge, our research is innovative to some extent as the diagnostic model for OA was developed without using a complete readymade scoring system. There are several semiquantitative scoring systems for OA, such as the Whole Organ MRI Score (Peterfy et al., 2004) and MRI OA Score (Hunter et al., 2011), which use manually obtained MRI features to display signs of the knee joint. These systems were developed to improve diagnostic efficiency and are used as core ideas in radiomics research (Tack et al., 2018; Pedoia et al., 2019). The crux of this matter is that disease diagnosis requires a comprehensive evaluation of the patient’s symptoms, signs, and auxiliary imaging examinations. A single scoring system considers only the radiological scope of OA, which is not ideal for the diagnosis of clinical OA. In addition, we constructed radiomics based on the initial pathological changes in osteoarthritic bone marrow edema, which are of great significance for the prediction and early diagnosis of clinical OA.

Our study had certain limitations. First, As a single center retrospective study, our sample size is relatively small, so compared to the radiomics model, the AUC value of the nomogram model did not show significant advantage, making it necessary to conduct large-scale, multicenter studies in the future. Second, this study is a clinical retrospective study, and its results need to be validated in large-scale prospective randomized controlled trials. Finally, histological examination cannot be performed in this study; therefore, the relationship between radiomic features and bone marrow edema remains unclear, and these examinations should be conducted in future studies.

## Conclusion

Radiomics analysis using MRI-subchondral bone marrow edema is an efficient and useful method for the diagnosis of KOA. The three models all demonstrate good diagnostic ability for the presence or absence of knee osteoarthritis. The nomogram model based on radiomics signatures and clinical features exhibited favorable diagnostic performance, indicating its potential as an auxiliary diagnostic tool in future clinical applications. This will increase the clinical predictive and diagnostic ability of knee osteoarthritis.

## Data Availability

The original contributions presented in the study are included in the article/Supplementary Material, further inquiries can be directed to the corresponding author.

## References

[B1] Alliston T. Hernandez C. J. Findlay D. M. Felson D. T. Kennedy O. D. (2018). Bone marrow lesions in osteoarthritis: what lies beneath. J. Orthop. Res. 36 (7), 1818–1825. 10.1002/jor.23844 29266428 PMC8607515

[B2] Amin S. LaValley M. P. Guermazi A. Grigoryan M. Hunter D. J. Clancy M. (2005). The relationship between cartilage loss on magnetic resonance imaging and radiographic progression in men and women with knee osteoarthritis. Arthritis Rheum. 52, 3152–3159. 10.1002/art.21296 16200595

[B3] Burr D. B. Gallant M. A. (2012). Bone remodelling in osteoarthritis. Nat. Rev. Rheumatol. 8 (11), 665–673. 10.1038/nrrheum.2012.130 22868925

[B4] Chimenti M. S. Conigliaro P. Navarini L. Martina F. M. Peluso G. Birra D. (2020). Demographic and clinical differences between ankylosing spondylitis and non-radiographic axial spondyloarthritis: results from a multicentre retrospective study in the Lazio region of Italy. Clin. Exp. Rheumatol. 38 (1), 88–93. 31140397

[B5] Cui T. Liu R. Jing Y. Fu J. Chen J. (2023). Development of machine learning models aiming at knee osteoarthritis diagnosing: an MRI radiomics analysis. J. Orthop. Surg. Res. 18 (1), 375. 10.1186/s13018-023-03837-y 37210510 PMC10199595

[B6] Demehri S. Kasaeian A. Roemer F. W. Guermazi A. (2023). Osteoarthritis year in review 2022: imaging. Osteoarthr. Cartil. 31 (8), 1003–1011. 10.1016/j.joca.2023.03.005 PMC12224773 36924919

[B7] Deng K. W. Liu J. L. Chen H. A. Li H. Wei T. Hao Q. (2021). Correlation between cold pain of knee joint and subchondral bone marrow edema in patients with knee osteoarthritis. Zhongguo Gu Shang 34 (2), 165–169. 10.12200/j.issn.1003-0034.2021.02.014 33666006

[B8] Dong B. Kong Y. Zhang L. Qiang Y. (2017). Severity and distribution of cartilage damage and bone marrow edema in the patellofemoral and tibiofemoral joints in knee osteoarthritis determined by MRI. Exp. Ther. Med. 13 (5), 2079–2084. 10.3892/etm.2017.4190 28565811 PMC5443282

[B9] Driban J. B. Price L. L. LaValley M. P. Lo G. H. Zhang M. Harkey M. S. (2022). Novel framework for measuring whole knee osteoarthritis progression using magnetic resonance imaging. Arthritis Care Res. Hob. 74 (5), 799–808. 10.1002/acr.24512 PMC8631200 33202111

[B10] Fan T. Ruan G. Antony B. Cao P. Li J. Han W. (2021). The interactions between MRI-detected osteophytes and bone marrow lesions or effusion-synovitis on knee symptom progression: an exploratory study. Osteoarthr. Cartil. 29 (9), 1296–1305. 10.1016/j.joca.2021.06.008 34216729

[B11] Gong J. Pedoia V. Facchetti L. Link T. M. Ma C. B. Li X. (2016). Bone marrow edema-like lesions (BMELs) are associated with higher T1ρ and T2 values of cartilage in anterior cruciate ligament (ACL)-reconstructed knees: a longitudinal study. Quant. Imaging Med. Surg. 6 (6), 661–670. 10.21037/qims.2016.12.11 28090444 PMC5219965

[B12] Guermazi A. Roemer F. W. Hayashi D. (2011). Imaging of osteoarthritis: update from a radiological perspective. Curr. Opin. Rheumatol. 23, 484–491. 10.1097/BOR.0b013e328349c2d2 21760511

[B13] Haugen I. K. Slatkowsky-Christensen B. Bøyesen P. Sesseng S. van der Heijde D. Kvien T. K. (2016). MRI findings predict radiographic progression and development of erosions in hand osteoarthritis. Ann. Rheum. Dis. 75, 117–123. 10.1136/annrheumdis-2014-205949 25204463

[B14] Hawker G. A. (2019). Osteoarthritis is a serious disease. Clin. Exp. Rheumatol. 37 (5), 3–6. 31621562

[B15] Hirvasniemi J. Klein S. Bierma-Zeinstra S. Vernooij M. W. Schiphof D. Oei E. H. G. (2021). A machine learning approach to distinguish between knees without and with osteoarthritis using MRI-based radiomic features from tibial bone. Eur. Radiol. 31 (11), 8513–8521. 10.1007/s00330-021-07951-5 33884470 PMC8523397

[B16] Horga L. M. Hirschmann A. C. Henckel J. Fotiadou A. Di Laura A. Torlasco C. (2020). Prevalence of abnormal findings in 230 knees of asymptomatic adults using 3.0 T MRI. Skelet. Radiol. 49 (7), 1099–1107. 10.1007/s00256-020-03394-z PMC7237395 32060622

[B17] Hu Y. Chen X. Wang S. Jing Y. Su J. (2021). Subchondral bone microenvironment in osteoarthritis and pain. Bone Res. 9 (1), 20. 10.1038/s41413-021-00147-z 33731688 PMC7969608

[B18] Hunter D. J. Guermazi A. Lo G. H. Grainger A. J. Conaghan P. G. Boudreau R. M. (2011). Evolution of semi-quantitative whole joint assessment of knee OA: MOAKS (MRI Osteoarthritis Knee Score). Osteoarthrit Cartil. 19, 990–1002. 10.1016/j.joca.2011.05.004 PMC4058435 21645627

[B19] Joint Surgery Branch of the Chinese Orthopaedic Association. Chinese guideline for diagnosis and treatment of osteoarthritis (2021 edition). Chin. J. Orthop., 2021, 41(18): 1291–1314.

[B20] Kon E. Ronga M. Filardo G. Farr J. Madry H. Milano G. (2016). Bone marrow lesions and subchondral bone pathology of the knee. Knee Surg. Sports Traumatol. Arthrosc. 24 (6), 1797–1814. 10.1007/s00167-016-4113-2 27075892

[B21] Kostopoulos S. Boci N. Cavouras D. Tsagkalis A. Papaioannou M. Tsikrika A. (2023). Radiomics texture analysis of bone marrow alterations in MRI knee examinations. J. Imaging 9 (11), 252. 10.3390/jimaging9110252 37998099 PMC10672553

[B22] Koushesh S. Shahtaheri S. M. McWilliams D. F. Walsh D. A. Sheppard M. N. Westaby J. (2022). The osteoarthritis bone score (OABS): a new histological scoring system for the characterisation of bone marrow lesions in osteoarthritis. Osteoarthr. Cartil. 30 (5), 746–755. 10.1016/j.joca.2022.01.008 PMC9395274 35124198

[B23] Kumar V. Gu Y. Basu S. Berglund A. Eschrich S. A. Schabath M. B. (2012). Radiomics: the process and the challenges. Magn. Reson Imaging 30 (9), 1234–1248. 10.1016/j.mri.2012.06.010 22898692 PMC3563280

[B24] Kuttapitiya A. Assi L. Laing K. Hing C. Mitchell P. Whitley G. (2017). Microarray analysis of bone marrow lesions in osteoarthritis demonstrates upregulation of genes implicated in osteochondral turnover, neurogenesis and inflammation. Ann. Rheum. Dis. 76 (10), 1764–1773. 10.1136/annrheumdis-2017-211396 28705915 PMC5629942

[B25] Leydet-Quilici H. Le Corroller T. Bouvier C. Giorgi R. Argenson J. N. Champsaur P. (2010a). Advanced hip osteoarthritis: magnetic resonance imaging aspects and histopathology correlations. Osteoarthr. Cartil. 18 (11), 1429–1435. 10.1016/j.joca.2010.08.008 20727415

[B26] Leydet-Quilici H. Le Corroller T. Bouvier C. Giorgi R. Argenson J. N. Champsaur P. (2010b). Advanced hip osteoarthritis: magnetic resonance imaging aspects and histopathology correlations. Osteoarthr. Cartil. 18 (11), 1429–1435. 10.1016/j.joca.2010.08.008 20727415

[B27] Li W. Feng J. Zhu D. Xiao Z. Liu J. Fang Y. (2023). Nomogram model based on radiomics signatures and age to assist in the diagnosis of knee osteoarthritis. Exp. Gerontol. 171, 112031. 10.1016/j.exger.2022.112031 36402414

[B28] Li X. Chen W. Liu D. Chen P. Wang S. Li F. (2024). Pathological progression of osteoarthritis: a perspective on subchondral bone. Front. Med. 2024. 10.1007/s11684-024-1061-y 38619691

[B29] Li Y. Li J. Zhu Z. Cao P. Han W. Ruan G. (2022). Signal intensity alteration and maximal area of pericruciate fat pad are associated with incident radiographic osteoarthritis: data from the osteoarthritis initiative. Eur. Radiol. 32, 489–496. 10.1007/s00330-021-08193-1 34327582

[B30] Lo G. H. McAlindon T. E. Niu J. Zhang Y. Beals C. Dabrowski C. (2009). Bone marrow lesions and joint effusion are strongly and independently associated with weight-bearing pain in knee osteoarthritis: data from the osteoarthritis initiative. Osteoarthr. Cartil. 17 (12), 1562–1569. 10.1016/j.joca.2009.06.006 PMC2787856 19583959

[B31] Luo P. Yuan Q. L. Yang M. Wan X. Xu P. (2023). The role of cells and signal pathways in subchondral bone in osteoarthritis. Bone Jt. Res. 12 (9), 536–545. 10.1302/2046-3758.129.BJR-2023-0081.R1 PMC10484649 37678837

[B32] Muratovic D. Findlay D. M. Cicuttini F. M. Wluka A. E. Lee Y. R. Edwards S. (2019). Bone marrow lesions in knee osteoarthritis: regional differences in tibial subchondral bone microstructure and their association with cartilage degeneration. Osteoarthr. Cartil. 27 (11), 1653–1662. 10.1016/j.joca.2019.07.004 31306782

[B33] Pedoia V. Norman B. Mehany S. N. Bucknor M. D. Link T. M. Majumdar S. (2019). 3D convolutional neural networks for detection and severity staging of meniscus and PFJ cartilage morphological degenerative changes in osteoarthritis and anterior cruciate ligament subjects. J. Magn. Reson. Imag. JMRI 49, 400–410. 10.1002/jmri.26246 PMC6521715 30306701

[B34] Peng Z. Sun H. Bunpetch V. Koh Y. Wen Y. Wu D. (2021). The regulation of cartilage extracellular matrix homeostasis in joint cartilage degeneration and regeneration. Biomaterials 268, 120555. 10.1016/j.biomaterials.2020.120555 33285440

[B35] Perry T. A. Parkes M. J. Hodgson R. Felson D. T. O'Neill T. W. Arden N. K. (2019). Effect of Vitamin D supplementation on synovial tissue volume and subchondral bone marrow lesion volume in symptomatic knee osteoarthritis. BMC Musculoskelet. Disord. 20 (1), 76. 10.1186/s12891-019-2424-4 30764805 PMC6376763

[B36] Perry T. A. Parkes M. J. Hodgson R. J. Felson D. T. Arden N. K. O'Neill T. W. (2020). Association between Bone marrow lesions and synovitis and symptoms in symptomatic knee osteoarthritis. Osteoarthr. Cartil. 28 (3), 316–323. 10.1016/j.joca.2019.12.002 PMC10536782 31877381

[B37] Peterfy C. G. Guermazi A. Zaim S. Tirman P. F. Miaux Y. White D. (2004). Whole-organ magnetic resonance imaging score (WORMS) of the knee in osteoarthritis. Osteoarthrit Cartil. 12, 177–190. 10.1016/j.joca.2003.11.003 14972335

[B38] Quicke J. G. Conaghan P. G. Corp N. Peat G. (2022). Osteoarthritis year in review 2021: epidemiology and therapy. Osteoarthr. Cartil. 30, 196–206. 10.1016/j.joca.2021.10.003 34695571

[B39] Tack A. Mukhopadhyay A. Zachow S. (2018). Knee menisci segmentation using convolutional neural networks: data from the Osteoarthritis Initiative. Osteoarthr. Cartil. 26 (5), 680–688. 10.1016/j.joca.2018.02.907 29526784

[B40] Wang W. Ye R. Xie W. Zhang Y. An S. Li Y. (2022). Roles of the calcified cartilage layer and its tissue engineering reconstruction in osteoarthritis treatment. Front. Bioeng. Biotechnol. 10, 911281. 10.3389/fbioe.2022.911281 36131726 PMC9483725

[B41] Wilson A. J. Murphy W. A. Hardy D. C. Totty W. G. (1988). Transient osteoporosis: transient bone marrow edema? Radiology 167 (3), 757–760. 10.1148/radiology.167.3.3363136 3363136

[B42] Xue Z. Wang L. Sun Q. Xu J. Liu Y. Ai S. (2022). Radiomics analysis using MR imaging of subchondral bone for identification of knee osteoarthritis. J. Orthop. Surg. Res. 17 (1), 414. 10.1186/s13018-022-03314-y 36104732 PMC9476345

[B43] Ye Q. He D. Ding X. Wang Y. Wei Y. Liu J. (2023). Quantitative evaluation of the infrapatellar fat pad in knee osteoarthritis: MRI-based radiomic signature. BMC Musculoskelet. Disord. 24 (1), 326. 10.1186/s12891-023-06433-7 37098523 PMC10127010

[B44] Yunus M. H. M. Nordin A. Kamal H. (2020). Pathophysiological perspective of osteoarthritis. Med. Kaunas. 56 (11), 614. 10.3390/medicina56110614 PMC7696673 33207632

[B45] Zhang H. Wang L. Cui J. Wang S. Han Y. Shao H. (2023a). Maintaining hypoxia environment of subchondral bone alleviates osteoarthritis progression. Sci. Adv. 9 (14), eabo7868. 10.1126/sciadv.abo7868 37018403 PMC10075992

[B46] Zhang J. Chen S. Chen W. Huang Y. Lin R. Huang M. (2018). Ultrastructural change of the subchondral bone increases the severity of cartilage damage in osteoporotic osteoarthritis of the knee in rabbits. Pathol. Res. Pract. 214 (1), 38–43. 10.1016/j.prp.2017.11.018 29263013

[B47] Zhang S. Li T. Feng Y. Zhang K. Zou J. Weng X. (2023b). Exercise improves subchondral bone microenvironment through regulating bone-cartilage crosstalk. Front. Endocrinol. (Lausanne) 14, 1159393. 10.3389/fendo.2023.1159393 37288291 PMC10242115

[B48] Zhang W. Doherty M. Peat G. Bierma-Zeinstra M. A. Arden N. K. Bresnihan B. (2010). EULAR evidence-based recommendations for the diagnosis of knee osteoarthritis. Ann. Rheum. Dis. 69, 483–489. 10.1136/ard.2009.113100 19762361

