# Supplementary material for: Radiomics analysis using magnetic resonance imaging of bone marrow edema for diagnosing knee osteoarthritis
Source: Front Bioeng Biotechnol. 2024 Jun 12;12:1368188. doi: 10.3389/fbioe.2024.1368188 (PMC11199411; doi:10.3389/fbioe.2024.1368188)
Supplement: Supplementary file 1 [file Table4.doc]

Hosmer−Lemeshow H test

| Clinic Signature | Rad Signature | Nomogram |
| --- | --- | --- |
| 0.995 | 0.124 | 0.282 |
| 0.001 | 0.213 | 0.267 |
