# Supplementary material for: Radiomics analysis using magnetic resonance imaging of bone marrow edema for diagnosing knee osteoarthritis
Source: Front Bioeng Biotechnol. 2024 Jun 12;12:1368188. doi: 10.3389/fbioe.2024.1368188 (PMC11199411; doi:10.3389/fbioe.2024.1368188)
Supplement: Supplementary file 2 [file Table3.doc]

Delong test

| **Nomogram Vs Clinic** | **Nomogram Vs Rad** | **cohort** |
| --- | --- | --- |
| ＜0.001 | 0.851 | Train |
| ＜0.001 | 0.942 | Test |
