# Supplementary figures and images for: Radiomics analysis using magnetic resonance imaging of bone marrow edema for diagnosing knee osteoarthritis
Source: Front Bioeng Biotechnol. 2024 Jun 12;12:1368188. doi: 10.3389/fbioe.2024.1368188 (PMC11199411; doi:10.3389/fbioe.2024.1368188)

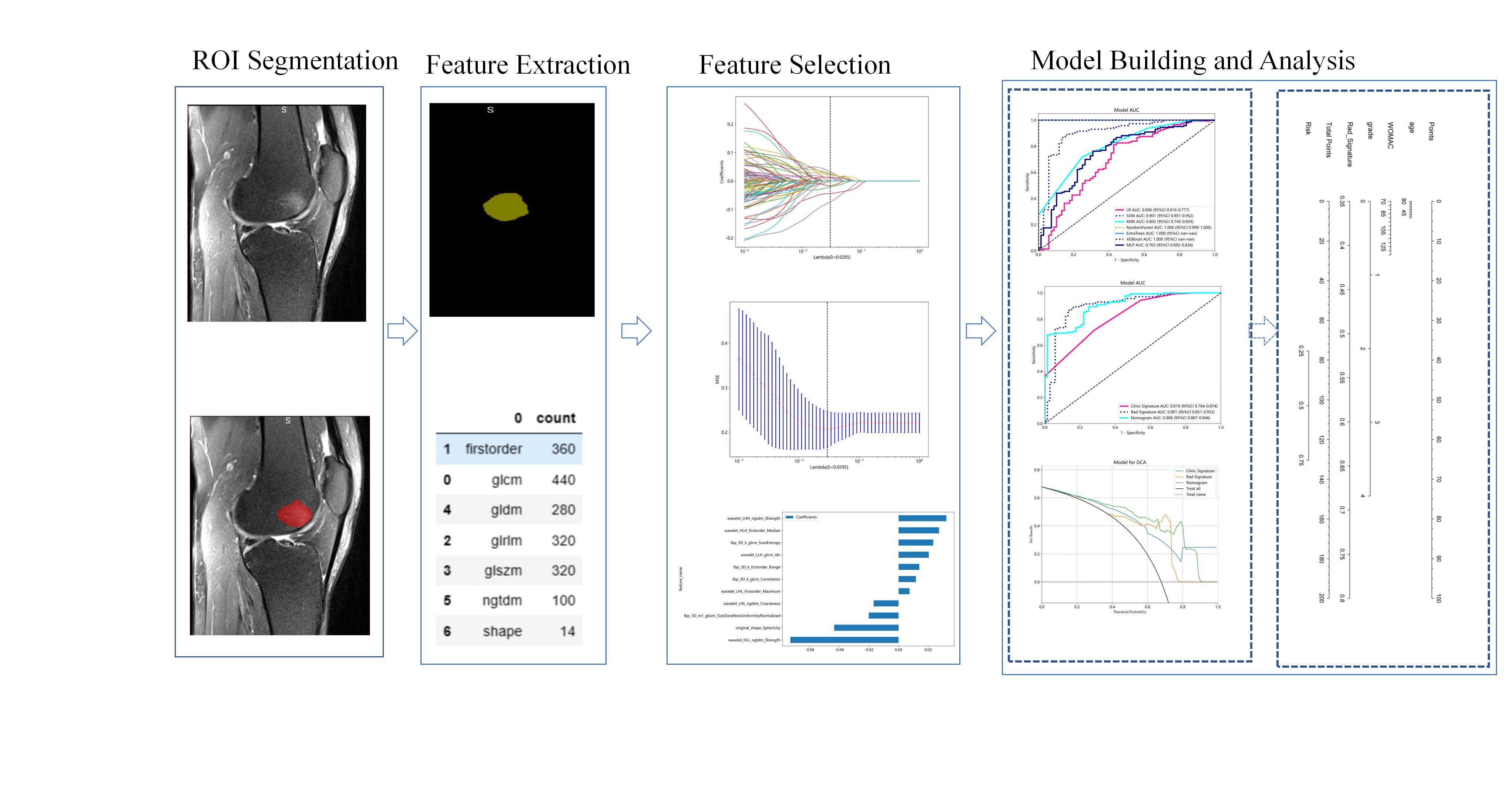

Supplement: Supplementary file 3 [file Image1.JPEG]
