# Supplementary material for: Radiomics analysis using magnetic resonance imaging of bone marrow edema for diagnosing knee osteoarthritis
Source: Front Bioeng Biotechnol. 2024 Jun 12;12:1368188. doi: 10.3389/fbioe.2024.1368188 (PMC11199411; doi:10.3389/fbioe.2024.1368188)
Supplement: Supplementary file 4 [file Table1.doc]

label = 0.6721854304635762 + +0.014005 * lbp_3D_k_firstorder_Range +0.011701 * lbp_3D_k_glcm_Correlation +0.023467 * lbp_3D_k_glcm_SumEntropy -0.020170 * lbp_3D_m1_glszm_SizeZoneNonUniformityNormalized -0.043521 * original_shape_Sphericity +0.027335 * wavelet_HLH_firstorder_Median -0.073228 * wavelet_HLL_ngtdm_Strength +0.032327 * wavelet_LHH_ngtdm_Strength +0.007426 * wavelet_LHL_firstorder_Maximum -0.016842 * wavelet_LHL_ngtdm_Coarseness +0.020427 * wavelet_LLH_glcm_Idn
