# Supplementary material for: Radiomics analysis using magnetic resonance imaging of bone marrow edema for diagnosing knee osteoarthritis
Source: Front Bioeng Biotechnol. 2024 Jun 12;12:1368188. doi: 10.3389/fbioe.2024.1368188 (PMC11199411; doi:10.3389/fbioe.2024.1368188)
Supplement: Supplementary file 5 [file Table2.doc]

Comparative analysis of different radiomics models

| **model_name** | **Accuracy** | **AUC** | **95% CI** | **Sensitivity** | **Specificity** | **PPV** | **NPV** | **Precision** | **Recall** | **F1** | **Threshold** | **Task** |
| --- | --- | --- | --- | --- | --- | --- | --- | --- | --- | --- | --- | --- |
| LR | 0.725 | 0.696 | 0.6158 - 0.7769 | 0.937 | 0.279 | 0.732 | 0.679 | 0.732 | 0.937 | 0.822 | 0.639 | label-train |
| LR | 0.747 | 0.822 | 0.7353 - 0.9088 | 0.900 | 0.452 | 0.761 | 0.700 | 0.761 | 0.900 | 0.824 | 0.621 | label-test |
| SVM | 0.768 | 0.901 | 0.8512 - 0.9518 | 0.993 | 0.894 | 0.747 | 0.952 | 0.747 | 0.993 | 0.853 | 0.708 | label-train |
| SVM | 0.681 | 0.841 | 0.7589 - 0.9239 | 0.950 | 0.861 | 0.687 | 0.625 | 0.687 | 0.950 | 0.797 | 0.665 | label-test |
| KNN | 0.758 | 0.802 | 0.7455 - 0.8594 | 0.937 | 0.382 | 0.761 | 0.743 | 0.761 | 0.937 | 0.840 | 0.800 | label-train |
| KNN | 0.736 | 0.745 | 0.6444 - 0.8454 | 0.917 | 0.387 | 0.743 | 0.706 | 0.743 | 0.917 | 0.821 | 0.800 | label-test |
| RandomForest | 0.995 | 1.000 | 0.9995 - 1.0000 | 1.000 | 0.985 | 0.993 | 1.000 | 0.993 | 1.000 | 0.997 | 0.600 | label-train |
| RandomForest | 0.725 | 0.748 | 0.6390 - 0.8578 | 0.783 | 0.613 | 0.797 | 0.594 | 0.797 | 0.783 | 0.790 | 0.600 | label-test |
| ExtraTrees | 1.000 | 1.000 | 1.0000 - 1.0000 | 1.000 | 1.000 | 1.000 | 1.000 | 1.000 | 1.000 | 1.000 | 1.000 | label-train |
| ExtraTrees | 0.780 | 0.802 | 0.7067 - 0.8965 | 0.867 | 0.613 | 0.812 | 0.704 | 0.812 | 0.867 | 0.839 | 0.600 | label-test |
| XGBoost | 0.991 | 1.000 | 1.0000 - 1.0000 | 1.000 | 0.971 | 0.986 | 1.000 | 0.986 | 1.000 | 0.993 | 0.680 | label-train |
| XGBoost | 0.747 | 0.796 | 0.7050 - 0.8874 | 0.817 | 0.613 | 0.803 | 0.633 | 0.803 | 0.817 | 0.810 | 0.648 | label-test |
| MLP | 0.716 | 0.763 | 0.6922 - 0.8342 | 0.951 | 0.221 | 0.720 | 0.682 | 0.720 | 0.951 | 0.819 | 0.672 | label-train |
| MLP | 0.703 | 0.796 | 0.7033 - 0.8891 | 0.917 | 0.290 | 0.714 | 0.643 | 0.714 | 0.917 | 0.803 | 0.727 | label-test |
